# Association between cytochrome P450 2C19 polymorphism and clinical outcomes in clopidogrel-treated Uygur population with acute coronary syndrome: a retrospective study

**DOI:** 10.1186/s12872-021-02201-4

**Published:** 2021-08-12

**Authors:** Luhai Yu, Tingting Wang, Huidong Bai, Weijiang Zhu, Yanju Li, Jianhua Wu, Wenli Liu, Li Sun, Aiping Yu, Hongjian Li

**Affiliations:** 1 Department of Pharmacy, People’s Hospital of Xinjiang Uygur Autonomous Region, No, 91 Tianchi Road, Tianshan District, Urumqi, 830001 Xinjiang China; 2 Institute of Clinical Pharmacy, People’s Hospital of Xinjiang Uygur Autonomous Region, No. 91 Tianchi Road, Tianshan District, Urumqi, 830001 Xinjiang China; 3 Dean’s Office, People’s Hospital of Xinjiang Uygur Autonomous Region, No. 91 Tianchi Road, Tianshan District, Urumqi, 830001 Xinjiang China

**Keywords:** Clopidogrel, *CYP2C19*, Acute coronary syndrome, Uygur, Major adverse cardiovascular events, Bleeding

## Abstract

**Background:**

Acute coronary syndrome (ACS) has become a vital disease with high mortality in the Uygur populations. Clopidogrel plays an important role in reducing the risk of recurrent cardiovascular events after ACS; however, it is a prodrug that requires biotransformation by cytochrome *P450* (*CYP450*).

**Objectives:**

To determine the effect of genetic polymorphisms in CYP2C19*2, *3, and *17, and along with clinical, demographic factors, on variation in response to clinical outcomes in Uygur patients.

**Methods:**

A total of 351 patients with ACS were treated with clopidogrel and aspirin for at least 12 months; we recorded major adverse cardiovascular events (MACE) or bleeding within 1 year. Multivariable logistic regression analyses were carried out to identify factors associated with MACE or bleeding.

**Results:**

We analyze risk factors include age, BMI (body mass index), smoking, alcohol intake, NSTEMI (non-ST-segment elevation myocardial infarction), hypertension, dyslipidemia, concomitant medication, CYP2C19*2 carriers, CYP2C19*17 carriers and metabolizer phenotype. CYP2C19*2 carriers had an odds of having MACE of 2.51 (95% CI: 1.534–4.09) compared with noncarriers (*P* < .001). However, no factors were significantly associated with bleeding (*P* > 0.05).

**Conclusion:**

The CYP2C19*2 gene polymorphism contributes to the risk of MACE in dual clopidogrel—treated Uygur population with ACS with or without PCI (percutaneous coronary intervention). These data may provide valuable insights into the genetic polymorphisms affecting clopidogrel metabolism among minority groups in China.

## Background

Acute coronary syndromes (ACS) are triggered by fissuring or rupture of an atheromatous plaque in the coronary arterial wall. This stimulates a thrombotic response causing variable obstruction to flow in the coronary arterial lumen with downstream ischaemic myocardial injury [1]. ACS is a life-threatening disease that is becoming the leading cause of morbidity and mortality in developing countries. Clopidogrel is the most widely prescribed purinergic receptor (P2Y12) inhibitor. It is an antiplatelet drug that, when administered with aspirin, has been shown to reduce the risk for cardiovascular events after ACS or a percutaneous coronary intervention (PCI) [2]. Clopidogrel is a prodrug requiring cytochrome P450 (*CYP*) for biotransformation into its active thiol metabolite. Initial clopidogrel pharmacogenetic studies examined genetic variations in *CYP* enzymes and associated these genetic variants with active metabolite levels. The most important of these is cytochrome P450, family 2, subfamily C, polypeptide 19 (*CYP2C19*), which metabolize clopidogrel to its active form [3].

The *CYP2C19* gene maps to the long arm of chromosome 10 (10q24) and it encodes a 490-amino-acid protein predominantly expressed in the liver and, to a lesser extent, in the small intestine. CYP2C19*2 results from a guanine (G) to adenine (A) transition at position 681 in exon5 (rs4244285), producing an aberrant splice site and it represents the most frequent *CYP2C19* defect [4], with the most common allele frequencies of 29–35% in Asians. Another nonfunctional allele CYP2C19*3, in which a guanine (G) to adenine (A) transition at position 636 in exon4 (rs4986893), results in a premature stop codon, and therefore nonfunctional protein [5, 6]. CYP2C19*3 is also relatively common in Asian populations (up to 10% are carriers) [7]. Patients with loss-of-function (LOF) allele variants (CYP2C19*2 and CYP2C19*3) are at risk for thromboembolic events [8]. The CYP2C19*17 allele was previously reported to be associated with high CYP2C19 activity. CYP2C19*17 is a -806 C > T single nucleotide polymorphism (with a cytosine (C) to thymine (T) transition) that causes specific nuclear protein binding to the 5'-flanking region. This binding results in increased gene transcription and high enzyme activities [9]. The *CYP2C19* gain-of-function allele (*17) is associated with increased catalytic activity [10]. In addition, polymorphisms of *CYP2C19* are known to vary considerably according to ethnicity [11, 12]. Approximately 25 genetic variants in the exonic region of the *CYP2C19* have been identified [4]. Other *CYP2C19* gene variants that lead to loss of function are relatively rare, typically below 1% [7]. Therefore, in our study, we determined the effect of genetic polymorphisms in CYP2C19*2, *3, and *17 on variation in response to clinical outcomes in Uygur ACS patients.

In 2012, the morbidity of coronary artery disease in the Uygur population of Xinjiang was 24.2%, much higher than the national average (7.2%) [13]. Polymorphisms of the *CYP2C19* gene and clinical factors are strong predictors of cardiovascular outcomes for patients with acute coronary syndrome treated with oral clopidogrel [14, 15]. Nevertheless, very little is known about the influence of such factors in Uygur populations. Therefore, we determined the long-term impact of *CYP2C19* polymorphisms on the risk for major adverse cardiac events (MACE) and bleeding in Uygur patients with ACS treated with clopidogrel.

## Methods

### Study population

We designed an observational case–control study to identify genetic and clinical factors associated with cardiovascular outcomes among Uygur patients with ACS. Patients presenting to People’s Hospital of Xinjiang Uygur Autonomous Region between July 5, 2014 and November 16, 2019 were considered for enrollment in our study. Eligible patients had a history of ACS (unstable angina or ST- segment elevation or non-ST- segment elevation myocardial infarction) or revascularization (any PCI or coronary artery bypass grafting). All patients should received a loading dose of clopidogrel 300 mg and aspirin 300 mg, followed by a 100 mg/day maintenance dose of aspirin and 75 mg/day of clopidogrel for > 1 year. The main exclusion criteria included the following: (1) history of bleeding and haemorrhagic disease; (2) significant valvular disease; (3) severe hepatic or renal dysfunction; (4) New York Heart Association (NYHA) grade IV heart failure; (5) have history of intermarriage with other ethnic groups within 3 generations.

### Ethical approval of the study protocol

The purpose and experimental procedures of the study were explained to all alive patients and legally authorized representative of one deceased patient, who gave informed written consent prior to the study. All patients or their authorized representative explicitly provided permission for genotyping as well as for collection of relevant clinical data. The study was conducted according to the standards of the Declaration of Helsinki and was approved by The Ethics Committees of People’s Hospital of Xinjiang Uygur Autonomous Region (approval number: 2014063).

### Blood sampling and genotyping

Blood samples were obtained from a peripheral vein and were collected in 4 mL vacuum tubes containing EDTA (BD). Samples were stored at − 20 °C until analysis. Genomic DNA was extracted from whole blood samples using the Puregene Blood Core Kit (Huaxia Times, China). CYP2C19*2 (681*G* > *A*, rs4244285), CYP2C19*3 (636*G* > *A*, rs4986893), CYP2C19*17 (-806*C* > *T*, rs12248560) were genotyped according to the manufacturer’s instructions using sequencing by hybridization (Realtime qPCR, Xi’an Tianlong Science & Technology Co Ltd, China). The blood sampling and genotyping were the same as our previous study [12].

### Outcomes and follow-up

Follow-ups were made mainly via outpatient interviews after discharge. Telephone interviews were done for the patients who lacked a medical record. Outcomes include 1) The occurrence of a MACE, defined as the all-cause death, nonfatal myocardial infarction (MI), unplanned target vessel revascularization (TVR), or stent thrombosis. All deaths were considered as cardiovascular deaths unless a clear non-cardiovascular cause was demonstrated. 2) Combined non-coronary artery bypass graft (CABG)-related bleeding. For subjects without a clinical event, follow-up was censored at the last clinic visit after 12 months of taking clopidogrel and aspirin.

### Case–control groups and CYP2C19 genotype-defined clopidogrel metabolic groups

Within this cohort of patients, efficacy controls were defined as patients who did not experience any MACE during12 months of antiplatelet therapy. Efficacy cases were defined as patients who experienced a MACE event within 12 months; Safety controls were defined as patients who did not experience any bleeding during12 months of antiplatelet therapy. Safety cases were defined as patients who experienced a bleeding event within 12 months.

Patients were categorized by genotype-defined clopidogrel metabolic groups based on CYP2C19*2, *3, and *17 genotypes, according to the Dutch Pharmacogenetics Working Group guidelines for clopidogrel and *CYP2C19*. Patients with at least 1 CYP2C19*2 or CYP2C19*3 allele variant were classified as loss-of-function allele carriers. Those with at least 2 CYP2C19*2 or CYP2C19*3 allele variants (*2/ *2,*2/ *3, or *3/ *3) were classified as poor metabolizers (PMs). Patients with 1 CYP2C19*2 or CYP2C19*3 allele variant (*1/ *2, *1/ *3), or 1 CYP2C19*17 allele variant with 1 (*2 or *3) allele variant (*2/ *17 or *3/ *17) were classified as intermediate metabolizers (IMs). Patients without a *2, *3, or *17 allele variant (*1/ *1) were classified as extensive metabolizers (EMs). Patients with at least 1*17 allele variant (*1/ *17 or *17/ *17) were classified as ultra-metabolizers (UMs). The *CYP2C19* genotype-defined clopidogrel metabolic groups were the same as our previous study [12].

### Statistical analysis

Continuous variables were expressed as mean values with standard deviations (SD). Categorical variables were expressed as patient numbers and percentages. The distributions of genotypes were assessed for deviation from the Hardy–Weinberg equilibrium (HWE) using the chi-square test. We compared quantitative dependent factors between the case and the control groups using one-way analysis of variance (ANOVA). Chi-square test or Fisher exact tests were used to compare the allele and genotype frequencies between cases and controls. A two-sided P value of less than 0.05 was used to indicate statistical significance. Multiple logistic regression analysis was used to identify factors associated with MACE or bleeding events. Results from the logistic regression analysis were described as odds ratios (ORs) with 95% confidence intervals (CIs). All statistical analyses were carried out using the SPSS 19.0 (version 4.0.100.1124, SPSS Inc).

## Results

### Baseline demographics, clinical characteristics

A total of 351 Uygur ACS patients were successfully enrolled in the study who were treated with clopidogrel and aspirin during 1 year of clinical follow-up. The clinical characteristics include sex, age, body mass index (BMI), alcohol intake, smoking, blood pressure, blood lipids level, transaminase, creatinine, uric acid, blood glucose, clinical presentation, final treatment, comorbidities, and concomitant medication. The mean age was 58.2 ± 9.0 years (range: 31–78 years); 79.2% of patients were male, with a mean BMI of 28.3 ± 3.8 kg/m^2^ and 78.6% underwent a PCI. Approximately 40.2% had a history of smoking; 51.6% presented with unstable angina; 16.0% presented with non-ST-segment elevation myocardial infarction (NSTEMI); and 32.5% with ST-segment elevation myocardial infarction. Hypertension and dyslipidemia were highly prevalent, at 56.1% and 77.5%, respectively. Most patients were treated with statins, β-blockers, angiotensin receptor blocker (ARB) or angiotensin antagonist inhibitor (ACEI), and 45.0% with proton pump inhibitor. These data are displayed in Table 1.

**Table 1:** Baseline demographics and characteristics of the study population

Characteristics and clinical outcomes	Total (n = 351)	MACE (n = 101)	No-MACE (n = 250)	*P* value	Bleeding (n = 18)	No-bleeding (n = 333)	*P* value
Male sex, n (%)	278 (79.2)	82 (81.2)	196 (78.4)	0.56	13 (72.2)	265 (79.6)	0.454
Age(years, mean ± SD)	58.2 ± 9.0	58.2 ± 8.5	58.3 ± 9.3	0.935	58.0 ± 8.6	58.2 ± 9.1	0.912
BMI (kg/m^2^, mean ± SD)	28.3 ± 3.8	28.7 ± 3.4	28.4 ± 5.3	0.649	26.8 ± 3.7	28.3 ± 3.8	0.094
Alcohol intake, n (%)	65 (18.5)	14 (13.9)	51 (20.4)	0.153	2 (11.1)	63 (18.9)	0.544
Smoking, n (%)	141 (40.2)	33 (32.7)	108 (43.2)	0.069	6 (33.3)	135 (40.5)	0.544
SBP (mmHg, mean ± SD)	133.3 ± 21.7	134.3 ± 22.2	132.9 ± 21.5	0.575	129.3 ± 19.4	135.5 ± 22.3	0.136
DBP (mmHg, mean ± SD)	80.2 ± 13.4	80.2 ± 13.9	80.3 ± 12.3	0.772	80.7 ± 16.0	80.2 ± 13.3	0.668
TG (mg/dL)	1.67 ± 0.70	1.72 ± 0.77	1.55 ± 0.48	0.148	1.46 ± 0.39	1.68 ± 0.71	0.375
LDL-C (mg/dL)	2.52 ± 0.89	2.41 ± 0.89	2.57 ± 0.57	0.126	2.54 ± 0.70	2.52 ± 0.90	0.943
HDL-C (mg/dL)	0.87 ± 0.23	0.85 ± 0.18	0.88 ± 0.25	0.314	0.92 ± 0.19	0.88 ± 0.24	0.509
TC (mg/dL)	4.03 ± 1.06	3.91 ± 1.0	4.07 ± 1.08	0.234	4.13 ± 0.97	4.0 ± 1.07	0.649
ALT (IU/L)	32.5 ± 13.6	29.5 ± 11.6	33.6 ± 15.3	0.206	24.5 ± 6.6	32.6 ± 17.8	0.222
AST (IU/L)	36.9 ± 15.5	39.2 ± 16.5	35.9 ± 12.9	0.622	27.5 ± 13.7	37.1 ± 21.9	0.478
Creatinine (μmol/L)	72.1 ± 17.9	71.7 ± 20.5	72.3 ± 16.8	0.778	78.4 ± 23.5	71.9 ± 17.4	0.134
UA (μmol/ L)	325.0 ± 58.8	323.4 ± 57.5	325.6 ± 59.5	0.831	356.5 ± 58.1	325.2 ± 46.5	0.148
BG (mmol/L)	6.49 ± 2.86	6.37 ± 2.47	6.53 ± 3.01	0.655	5.98 ± 1.98	6.5 ± 2.92	0.483
Clinical presentation, n (%)							
Unstable angina	181 (51.6)	54 (53.5)	127 (50.8)	0.651	9 (50)	172 (51.7)	0.891
STEMI	114 (32.5)	35 (34.7)	79 (31.6)	0.58	4 (22.2)	110 (33.0)	0.443
NSTEMI	56 (16.0)	26 (25.7)	30 (12.0)	0.001*	3 (16.7)	53 (15.9)	1.0
Final treatment, n (%)							
PCI	276 (78.6)	80 (79.2)	196 (78.4)	0.867	8 (44.4)	268 (80.5)	0.001*
CABG	5 (1.4)	/	5 (2.0)	0.327	2 (11.1)	3 (0.9)	0.023*
Medical treatment only	70 (19.9)	21 (20.8)	49 (19.6)	0.8	8 (44.4)	62 (18.6)	0.008*
Comorbidities, n%							
Hypertension	197 (56.1)	57 (56.4)	140 (56.0)	0.941	9 (50)	190 (57.1)	0.556
Fatty liver	109 (31.1)	32 (31.7)	77 (30.8)	0.871	3 (16.7)	106 (31.8)	0.203
Diabetes mellitus	138 (39.3)	39 (38.6)	99 (39.6)	0.864	7 (38.9)	131 (39.3)	1.0
Dyslipidemia	272 (77.5)	77 (76.2)	195 (78)	0.72	13 (72.2)	259 (77.8)	0.583
History, n (%)							
Previous MI	77 (21.9)	22 (21.8)	55 (22.0)	0.964	2 (11.1)	75 (22.5)	0.382
Previous PCI	44 (12.50)	14 (13.9)	30 (12.0)	0.634	1 (5.6)	43 (12.9)	0.712
Previous CABG	5 (1.4)	1 (1.0)	4 (1.6)	1.0	/	5 (2.1)	1.0
Concomitant medication, n%							
Statin	339 (96.6)	99 (98.0)	240 (96.0)	0.521	18 (100)	321 (96.4)	1.0
PPI	158 (45.0)	46 (45.5)	112 (44.8)	0.899	9 (50)	149 (44.7)	0.662
CCB	85 (24.2)	24 (23.8)	61 (24.4)	0.9	6 (33.3)	79 (23.7)	0.354
β-Blocker	287 (81.8)	88 (87.1)	199 (79.6)	0.098	12 (66.7)	275 (82.6)	0.088
Diuretics	92 (26.2)	32 (31.7)	60 (24.0)	0.138	5 (27.8)	87 (26.1)	0.877
ARB or ACEI	266 (75.8)	73 (72.3)	193 (77.2)	0.330	10 (55.6)	257 (77.2)	0.036*

*BMI* body mass index, *SBP* systolic blood pressure, *DBP* diastolic blood pressure, *TG* triglycerides, *LDL-C* low density lipoprotein cholesterol, *HDL-C* high density lipoprotein cholesterol, *TC* total cholesterol, *ALT* glutamic-pyruvic transaminase, *AST* glutamic-oxalacetic transaminase, *UA* uric acid, *BG* blood glucose, *NSTEMI* non-ST-segment elevation myocardial infarction, *STEMI* ST-segment elevation myocardial infarction, *MACE* major adverse cardiac events, *MI* myocardial infarction, *PCI* percutaneous coronary intervention, *CABG* coronary artery bypass grafting, *ARB* angiotensin receptor blocker, *ACEI* angiotensin antagonist inhibitor, *CCB* calcium channel blocker, *PPI* proton pump inhibitor

*P < 0.05,* the difference of MACE vs. No-MACE or Bleeding vs. No-Bleeding group by χ^2^ test at 0.05

### Outcomes

There were 101 patients (28.8%) who suffered MACE during the 1-year follow-up period, including one (0.3%) characterized as an all-cause death; 57 patients suffered nonfatal MI (16.2%); 20 patients experienced stent thrombosis (5.7%); 35 patients were re-hospitalized for revascularization (10.0%) during the 1-year follow-up. Bleeding occurred in 18 patients (5.1%), including gingival bleeding, skin ecchymosis, gastrointestinal bleeding, fecal occult blood, and occult blood in the urine. All clinical outcomes are summarized in Table 2.

**Table 2:** Clinical outcomes of the study population

Clinical outcomes, n (%)	Total (n = 351)
MACE	101 (28.8)
Nonfatal MI	57 (16.2)
Stent thrombosis	20 (5.7)
Unplanned TVR	35 (10.0)
All-cause death	1 (0.3)
Bleeding	18 (5.1)

*MACE* major adverse cardiac events, *MI* myocardial infarction, *TVR* target vessel revascularization

### Clinical characteristics in MACE, no-MACE, bleeding, and no-bleeding patients

The no-MACE group consisted of 250 (71.2%) participants who did not experience MACE. Compared with the no-MACE group (12.0%), the MACE group had a significantly higher prevalence of NSTEMI (25.7%, *P* = 0.001). Other characteristics were similar between the two groups. There were 333 (94.9%) participants who did not experience any bleeding. Compared with the no-bleeding group, the bleeding group had a significantly higher frequency of definitive treatment of coronary artery bypass grafting and with medical treatment only (11.1% and 44.4%, respectively) than the non-bleeding group (0.9%, 18.6%, respectively,* P* < 0.05). Conversely, more participants underwent a PCI in the non-bleeding group (80.5%) than the bleeding group (44.4%, *P* < 0.05). ARB or ACEI use was more common in the non-bleeding group (77.2%). These data are displayed in Table 1.

### Distribution of CYP2C19 polymorphisms and metabolic groups in the study population

We genotyped the three genetic variants (CYP2C19*2, *3, and *17) associated with clinical effects and bleeding events associated with clopidogrel. The distribution of the genetic polymorphisms and *CYP2C19* genotype-defined clopidogrel metabolic groups in the overall MACE, no-MACE, bleeding, and no-bleeding patients is displayed in Table 3. All genetic variants achieved Hardy‐Weinberg equilibrium (*P* > 0.05). The MACE group had a higher CYP2C19*2 AA genotype frequency (37.6%) and higher *GA* genotype frequency (5.0%) than the no-MACE group, 20.8%, and 2.0%, respectively (*P* = 0.001). The frequencies of the *A* alleles were 23.8% in the MACE group and 12.4% in the no-MACE group (*P* < 0.001). The no-MACE group had a higher CYP2C19*17 T allele frequency (17.0%) than the MACE group (10.9%; *P* = 0.041). IMs in the no-MACE group and MACE group occurred at 33.7% and 29.2%, respectively. The frequencies of PMs in the no-MACE and MACE groups were 4.0% and 3.2%, respectively. The IMs and PMs were significantly higher in the no-MACE group than the MACE group (*P* < 0.05). By contrast, the frequency of UMs within the MACE group (13.9%) was significantly lower than in the no-MACE group (28.4%, *P* = 0.004). Neither the genetic polymorphisms nor metabolic groups differed significantly in frequency between the bleeding and the non-bleeding groups (*P* > 0.05). The genotyping results are displayed in Table 3.

**Table 3:** Distribution of *CYP2C19* polymorphisms in MACE, no-MACE, bleeding, and no-bleeding patients

Variables	Overall (n = 351)	MACE (n = 101)	No-MACE (n = 250)	*P* value	Bleeding (n = 18)	No-bleeding (n = 333)	*P* value
Polymorphisms, n (%)							
CYP2C19*2							
* GG*	251 (71.5)	58 (57.4)	193 (77.2)	0.001*	16 (88.9)	235 (70.6)	0.273
* GA*	90 (25.6)	38 (37.6)	52 (20.8)		2 (11.1)	88 (26.4)	
* AA*	10 (2.9)	5 (5.0)	5 (2.0)		/	10 (3.0)	
* A* allele	110 (15.7)	48 (23.8)	62 (12.4)	< 0.001*	2 (5.6)	108 (16.2)	0.139
* G* allele	692 (84.3)	154 (76.2)	438 (87.6)		34 (94.4)	558 (83.8)	
CYP2C19*3							
* GG*	328 (93.4)	91 (90.1)	237 (94.8)	0.107	16 (88.9)	312 (93.7)	0.333
* GA*	23 (6.6)	10 (9.9)	13 (5.2)		2 (11.1)	21 (6.3)	
* AA*	/	/	/		/	/	
* A* allele	23 (3.3)	10 (5.0)	13 (2.6)	0.113	2 (5.6)	21 (3.2)	0.332
* G* allele	679 (96.7)	192 (95.0)	487 (97.4)		34 (94.4)	645 (96.8)	
CYP2C19*17							
* CC*	252 (71.8)	81 (80.2)	171 (68.4)	0.08	10 (55.6)	242 (72.7)	0.139
* CT*	91 (25.9)	18 (17.8)	73 (29.2)		7 (38.9)	84 (25.2)	
* TT*	8 (2.3)	2 (2.0)	6 (2.4)		1 (5.6)	7 (2.1)	
* T* allele	107 (15.2)	22 (10.9)	85 (17.0)	0.041*	9 (25.0)	98 (14.7)	0.094
* C* allele	595 (84.8)	180 (89.1)	415 (83.0)		27 (75.0)	568 (85.3)	
Metabolizer Phenotype, n (%)							
EMs	147 (41.9)	37 (36.6)	110 (41.6)	0.205	7 (38.9)	140 (42.0)	0.792
IMs	107 (30.5)	43 (42.6)	64 (25.6)	0.002*	4 (22.2)	103 (30.9)	0.601
PMs	12 (3.4)	7 (6.9)	5 (2.0)	0.021*	/	12 (3.6)	1.0
UMs	85 (24.2)	14 (13.9)	71 (28.4)	0.004*	7 (38.9)	78 (23.4)	0.158

*MACE* major adverse cardiac events, *EMs* extensive metabolizers, *IMs* intermediate metabolizers, *PMs* poor metabolizers, *UMs* ultra-metabolizers; *P < 0.05,* the difference of MACE vs. no-MACE by χ^2^ test at 0.05

### Comparison of clinical backgrounds between MACE and no-MACE group of CYP2C19*2 carriers

There were 100 patients with the CYP2C19*2 A allele in total. The clinical backgrounds include sex, age, BMI, alcohol intake, smoking, blood pressure, blood lipids level, transaminase, creatinine, uric acid, blood glucose, clinical presentation, final treatment, comorbidities, and concomitant medication. There were no significant differences in any parameter at baseline between the MACE and no-MACE groups among CYP2C19*2 carriers (*P* > 0.05). The details are displayed in Table 4.

**Table 4:** Clinical backgrounds between MACE and no-MACE group of CYP2C19*2 carriers

Clinical backgrounds	Total (n = 100)	MACE (n = 43)	No-MACE (n = 57)	*P* value
Male sex, n (%)	89 (89.0)	40 (93.0)	49 (86.0)	0.343
Age(years, mean ± SD)	58.0 ± 8.8	58.5 ± 8.6	57.6 ± 9.0	0.633
BMI (kg/m^2^, mean ± SD)	28.2 ± 3.9	28.9 ± 3.3	27.7 ± 4.2	0.122
Alcohol intake, n (%)	18 (18.0)	7 (16.3)	11 (19.3)	0.697
Smoking, n (%)	42 (42.0)	18 (41.9)	24 (42.1)	0.980
SBP (mmHg, mean ± SD)	133.3 ± 21.7	137.2 ± 26.6	131.5 ± 24.8	0.174
DBP (mmHg, mean ± SD)	79.5 ± 13.7	80.1 ± 12.7	79.0 ± 14.5	0.664
TG (mg/dL)	1.65 ± 0.78	1.67 ± 0.56	1.64 ± 0.73	0.887
LDL-C (mg/dL)	2.50 ± 0.90	2.43 ± 0.93	2.57 ± 0.88	0.426
HDL-C (mg/dL)	0.85 ± 0.20	0.85 ± 0.16	0.86 ± 0.23	0.684
TC (mg/dL)	3.98 ± 1.01	3.94 ± 0.98	4.0 ± 1.04	0.731
ALT (IU/L)	35.8 ± 18.7	30.5 ± 11.9	40.0 ± 17.7	0.205
AST (IU/L)	39.7 ± 16.0	35.2 ± 15.6	43.1 ± 16.4	0.375
Creatinine (μmol/ L)	72.1 ± 19.0	71.9 ± 25.2	72.3 ± 12.4	0.926
UA (μmol/ L)	321.1 ± 61.9	320.0 ± 56.0	322.0 ± 66.8	0.914
BG (mmol/ L)	6.52 ± 2.61	6.79 ± 2.83	6.32 ± 2.45	0.375
Clinical presentation, n (%)				
Unstable angina	49 (49.0)	20 (46.5)	29 (50.9)	0.665
STEMI	32 (32.0)	13 (30.2)	19 (33.3)	0.742
NSTEMI	24 (24.0)	13 (30.2)	11 (19.3)	0.205
Final treatment, n (%)				
PCI	82 (82.0)	38 (88.4)	44 (77.2)	0.15
CABG	2 (2.0)	/	2 (3.5)	0.505
Medical treatment only	17 (17.0)	5 (11.6)	12 (21.1)	0.214
Comorbidities, n%				
Hypertension	57 (57.0)	32 (66.7)	30 (48.4)	0.067
Fatty liver	26 (26.0)	14 (32.6)	12 (21.1)	0.194
Diabetes mellitus	37 (37.0)	20 (46.5)	17 (29.8)	0.087
Dyslipidemia	83 (83.0)	37 (86.0)	46 (80.7)	0.481
History, n (%)				
Previous MI	23 (23.0)	9 (20.9)	14 (24.6)	0.669
Previous PCI	13 (13.0)	5 (11.6)	8 (14.0)	0.723
Previous CABG	1 (1.0)	/	1 (1.8)	1.0
Concomitant medication, n%				
Statin	96 (96.0)	41 (95.3)	55 (96.5)	1.0
PPI	44 (44.0)	16 (37.2)	28 (49.1)	0.235
CCB	26 (26.0)	12 (27.9)	14 (24.6)	0.706
β-Blocker	82 (82.0)	37 (86.0)	45 (78.9)	0.36
Diuretics	25 (25.0)	11 (25.6)	14 (24.6)	0.907
ARB or ACEI	77 (77.0)	33 (76.7)	44 (77.2)	0.958

*BMI* body mass index, *SBP* systolic blood pressure, *DBP* diastolic blood pressure, *TG* triglycerides, *LDL-C* low density lipoprotein cholesterol, *HDL-C* high density lipoprotein cholesterol, *TC* total cholesterol, *ALT* glutamic-pyruvic transaminase, *AST* glutamic-oxalacetic transaminase, *UA* uric acid, *BG* blood glucose, NSTEMI non-ST-segment elevation myocardial infarction, *STEMI* ST-segment elevation myocardial infarction, *MACE* major adverse cardiac events, *MI* myocardial infarction, *PCI* percutaneous coronary intervention, *CABG* coronary artery bypass grafting, *ARB* angiotensin receptor blocker, *ACEI* angiotensin antagonist inhibitor, *CCB* calcium channel blocker, *PPI* proton pump inhibitor

### The risk factors of MACE and bleeding

We analyzed risk factors for MACE and bleeding using by multivariate logistic regression analysis. The factors included age, BMI, smoking, alcohol intake, NSTEMI, hypertension, dyslipidemia, concomitant medication, CYP2C19*2 carriers, CYP2C19*17 carriers and metabolizer phenotype. In the analysis, CYP2C19*2 carriers was significantly associated with MACE. CYP2C19*2 carriers had an odds of having MACE of 2.51 (95% CI: 1.534–4.09) compared with noncarriers (*P* < 0.001). However, age, BMI, smoking, alcohol intake, NSTEMI, hypertension, dyslipidemia, concomitant medication, CYP2C19*17 carriers, and metabolizer phenotype were no longer significantly associated with MACE. By contrast, no factors were significantly associated with bleeding. All results are displayed in Table 5.

**Table 5:** The multiple logistics regression analysis of risk factors for MACE and Bleeding

Variables (%)	MACE (n = 101)	No-MACE (n = 250)	OR (95% CI)	*P* value	Bleeding (n = 18)	No-Bleeding (n = 333)	OR (95% CI)	*P* value
Age > 65	23 (22.8)	66 (26.4)	0.767 (0.423–1.389)	0.381	6 (33.3)	83 (24.9)	0.660 (0.203–2.150)	0.491
BMI > 26 kg/m^2^	81 (80.2)	178 (71.2)	1.436 (0.795–2.595)	0.230	9 (50)	250 (75.1)	2.615 (0.779–8.781)	0.12
Smoking	33 (32.7)	108 (43.2)	0.751 (0.418–1.351)	0.340	6 (33.3)	135 (40.5)	1.218 (0.321–4.611)	0.772
Alcohol intake	65 (18.5)	14 (13.9)	0.475 (0.491- 0.766)	0.491	2 (11.1)	63 (18.9)	1.894 (0.298–12.026)	0.498
NSTEMI	26 (25.7)	30 (12.0)	0.586 (0.238–1.443)	0.245	3 (16.7)	53 (15.9)	0.298 (0.074–1.196)	0.088
Hypertension	57 (56.4)	140 (56.0)	1.147 (0.671–1.963)	0.616	9 (50)	190 (57.1)	1.164 (0.323–4.193)	0.817
Dyslipidemia	77 (76.2)	195 (78)	1.029 (0.574–1.843)	0.924	13 (72.2)	259 (77.8)	1.761 (0.582–5.323)	0.316
Concomitant medication								
β-Blocker	88 (87.1)	199 (79.6)	1.552 (0.79–3.046)	0.202	12 (66.7)	275 (82.6)	2.181 (0.648–7.337)	0.208
ARB or ACEI	73 (72.3)	194 (77.6)	0.637 (0.351–1.155)	0.137	10 (55.6)	257 (77.2)	1.867 (0.534–6.531)	0.328
CYP2C19*2 carriers	43 (42.6)	57 (22.8)	2.51 (1.534–4.09)	< 0.001*	2 (11.1)	98 (29.4)	4.111 (0.440–37.414)	0.215
CYP2C19*17 carriers	20 (19.8)	79 (31.6)	1.084 (0.331–3.549)	0.893	8 (44.4)	93 (27.9)	0.171 (0.012–2.360)	0.187
Metabolizer phenotype								
IMs	43 (42.6)	64 (25.6)	1.829 (0.463–7.226)	0.389	4 (22.2)	103 (30.9)	1.17 (0.158–8.652)	0.878
PMs	7 (6.9)	5 (2.0)	3.643 (0.804–16.501)	0.094	/	12 (3.6)	/	/
UMs	14 (13.9)	71 (28.4)	0.477 (0.125–1.826)	0.192	7 (38.9)	78 (23.4)	0.227 (0.012–4.262)	0.322

BMI, body mass index; NSTEMI, non-ST-segment elevation myocardial infarction; ARB, angiotensin receptor blocker; ACEI, angiotensin antagonist inhibitor; MACE, major adverse cardiac events; IMs, intermediate metabolizers; PMs, poor metabolizers; UMs, ultra-metabolizers

*P < 0.05, * the difference of MACE *vs.* No-MACE group by χ^2^ test at 0.05

## Discussion

The Uygur population has a high prevalence of CHD. A major contributor is a high-fat diet and lifestyle associated with this community. Patients enrolled in this study showed high prevalences of hypertension, dyslipidemia, diabetes mellitus, and smoking. In Asian populations, obesity is defined as BMI > 26 kg/m^2^ [16]. The average BMI of the Uygur patients in this study was 28.3 ± 3.8 kg/m^2^. According to many recent estimates, advanced age is an independent risk factor of CHD, primarily attributed to compromised plasticity of vessels [17]. The mean age of our patients was 58.2 ± 9.0 years (range: 31–78 years), and 25.4% of these patients were > 65 years. Crimi et al. reported that cigarette smoking reduced platelet reactivity independently of clopidogrel treatment in patients with ACS [18]. Smoking is an important risk factor primarily because cigarette chemicals cause coronary inflammation [19], and 40.2% of our patients with a smoking history developed ACS. Furthermore, most of our subjects had at least one cardiovascular risk factor. Demographic characteristics of the study patients were according to the cardiovascular risk factors [20].

Clopidogrel is a therapy for ACS and emergent or elective PCI. Clopidogrel has no biological activity; it is metabolized into 15% active metabolite and 85% inactive metabolite by the P450 system. The polymorphic isoenzyme *CYP2C19* plays an essential role in genetic diversity [21]. There were 101 patients (28.8%) who suffered MACE during the 1-year follow-up in the current study. By comparison, we found that the MACE group had a significantly higher CYP2C19*2 AA genotype frequency and higher *GA* genotype frequency than the no-MACE group (*P* = 0.001). These findings suggest that *CYP2C19* is an independent predictor of the risk of MACE in Uygur patients, following several studies of Chinese patients undergoing PCI [15, 22]. It is well-established that patients carrying *CYP2C19* (LOF) alleles have a reduced capacity for clopidogrel bioactivation, impaired platelet inhibition, and a significantly higher risk of MACE when treated with clopidogrel compared with patients without a LOF allele [23]. This finding was demonstrated in the present study; the MACE group had a significantly higher CYP2C19*2 A allele frequency than the no-MACE group (*P* < 0.001).

Based on their abilities to metabolize *CYP2C19* substrates, individuals may be classified as EMs, IMs, PMs, or UMs [24–26]. *CYP2C19* IMs and PMs have significantly lower plasma concentrations of the active metabolite and diminished inhibition of platelet aggregation than EMs [27]. The prevalence of IMs and PMs is significantly higher in Asians, with about 57% of LOF allele carriers [28]. In the current study, about 35% of Uygur ACS patients carried an LOF allele (110 CYP2C19*2 carriers and 23 CYP2C19*3 carriers). Both IMs and PMs were significantly higher in the no-MACE group than in the MACE group (*P* < 0.05). This finding accords with a meta-analysis of 36,076 participants that found that IMs and PMs in Asian populations undergoing PCI and prescribed clopidogrel had a higher risk of MACE [29].

According to the characteristics and the distribution of *CYP2C19* polymorphisms, we analyzed risk factors for MACE and bleeding using multivariate logistic regression analysis. In multiple logistic regression analysis, only CYP2C19*2 carriers were significantly associated with MACE (OR: 2.51, 95% CI: 1.534–4.09). Shuldiner et al. reported that, in a healthy Amish population, the CYP2C19*2 allele was the primary genetic locus associated with diminished platelet inhibitory response to clopidogrel, and that the CYP2C19*2 allele accounted for 12% of the inter-patient variability in platelet response to clopidogrel [27]. Besides, we compared clinical backgrounds between MACE and no-MACE group of CYP2C19*2 carriers. The clinical backgrounds include sex, age, BMI, alcohol intake, smoking, blood pressure, blood lipids level, transaminase, creatinine, uric acid, blood glucose, clinical presentation, final treatment, comorbidities, and concomitant medication. There were no significant differences in clinical backgrounds between MACE and no-MACE groups of CYP2C19*2 carriers (*P* > 0.05). This suggests that logistic regression analysis did not have the influence of other clinical backgrounds.

Another study showed that *CYP2C19* UMs treated with clopidogrel exhibited increased active metabolite formation, inhibition of platelet aggregation, higher bleeding risk, and lower MACE risk [30]. CYP2C19*17 was linked to a superior response to clopidogrel but an increased risk of bleeding [31]. In the present study, bleeding occurred in 18 patients (5.1%) during the 1-year follow-up; this rate is consistent with that of Korean ACS patients after taking clopidogrel (5.3%) [32].

Although the UMs and CYP2C19*17 carriers did not contribute to the risk of bleeding events in our analysis, we believe that the results are less persuasive because of the limited sample size (only 18 patients bled). On the other hand, the frequency of UMs in the no-MACE group was significantly higher than the MACE group in Uygur patients (*P* = 0.004). This finding suggests that the frequency of the CYP2C19*17T allele in the bleeding group (25.0%) was higher than that in the non-bleeding group (14.7%), although not significantly (*P* > 0.05). Our previous report found that allele frequencies of the minor alleles of CYP2C19 *2, *3, and *17 differed significantly between Uygur and Han groups [12]. To our knowledge, this is the first study reporting the impact of the cytochrome P450 *2C19* polymorphism of MACE and bleeding to clopidogrel in Uygur patients with ACS.

Our study has the following limitations. First, only 351 Uygur ACS patients were assessed; thus, our findings regarding the association between the *CYP2C19* polymorphism and clinical outcomes of clopidogrel treatment should be validated in studies with larger samples. Second, because of the limitation of detection technology, we could not establish the value of *CYP2C19* genotyping combined with on-treatment platelet reactivity (platelet function test). Third, we obtained some MACE and bleeding information via telephone and therefore could not precisely document symptoms. Finally, gender is a nonmodifiable risk factor of ACS [33]; the varying prevalence of ACS results might result from the unbalanced gender distribution.

## Conclusion

The CYP2C19*2 gene polymorphism is an essential factor associated with MACE risk in the dual clopidogrel-treated Uygur population with ACS with or without PCI. These data provide valuable insights into the genetic polymorphisms affecting clopidogrel metabolism among minority groups. We aim to determine the most effective and safe individualized ACS therapies for various ethnic groups in Xinjiang.

## Data Availability

The datasets used and/or analysed during the current study available from the corresponding author on reasonable request.
